# RANZCP Position Statement 46: A missed opportunity to provide sophisticated guidance on asylum seeker and refugee policy

**DOI:** 10.1177/10398562241304929

**Published:** 2024-12-02

**Authors:** Jillian Spencer

**Affiliations:** 67568 Queensland Children’s Hospital, Brisbane, QLD, Australia

**Keywords:** immigration, advocacy, asylum seekers, refugees, detention

## Abstract

**Objective:**

To stimulate discussion on how the RANZCP can contribute more constructively to the debate over Australia’s immigration policies.

**Conclusions:**

Updated in March 2024, RANZCP Position Statement 46, titled: ‘The provision of mental health services for asylum seekers and refugees’, continues the College’s advocacy for a compassionate stance towards asylum seekers and refugees on the grounds of preventing or improving their mental health. College statements over the last decade have raised concerns about policies that are designed to deter boat arrivals; and recently, have endorsed the High Court’s NZYQ decision to mandate community release of detained non-Australian citizens deemed to have failed ‘the character test’ under the *Migration Act 1958 (Cth)*. The College appears to have avoided addressing public concern about how a high asylum seeker and refugee inflow may impact community cohesion and prosperity. RANZCP’s reputation will be enhanced by ensuring more extensive explication of reasoning and rebuttal of counter arguments for the position it has taken on this complex political issue.

## Historical background to Australia’s immigration policies

Australia’s immigration policies, particularly regarding asylum seekers and refugees, have been subject to vigorous public debate for over 30 years. Mandatory detention for unauthorised boat arrivals was first introduced by the Keating Labor Government and came into operation in 1994.^1^ This legislation allowed for potentially indefinite administrative detention until release through the visa grant or repatriation.

Boat arrivals became a major election issue in 2001 following a quadrupling of arrivals (see Table 1). In 2001, the ‘Tampa crisis’, as well as allegations of asylum seekers threatening to throw children overboard if not taken to Australia, was credited with the Howard Government retaining office in the 2001 federal election with a suite of measures termed ‘the Pacific Solution’.^2^ These measures included the excision of Christmas, Ashmore and Cartier Islands from Australia’s migration zone, thereby preventing people from activating Australia’s protection obligations.^1^ People arriving by boat were transported to facilities in Nauru or Papua New Guinea for asylum claim processing. Australia also introduced Temporary Protection Visas, enhanced coastal surveillance, asylum seeker boat turnbacks, no rights of family reunion for people arriving by boat and increased its engagement with transit countries to interrupt people smuggling operations.^3^

**Table 1.** Number of boats and number of people arriving by boat to Australia per year

Year	Number of boats	Number of people arriving
1989	1	26
1990	2	198
1991	6	214
1992	6	216
1993	3	81
1994	18	953
1995	7	237
1996	19	660
1997	11	339
1998	17	200
1999	86	3721
2000	51	2939
2001	43	5516
2002	1	1
2003	1	53
2004	1	15
2005	4	11
2006	6	60
2007	5	148
2008	7	161
2009	60	2726
2010	134	6555
2011	69	4565
2012	278	17204
2013	300	20587
2014	12	450
2015	11	217
2016	6	51
2017	3	60
2018	2	24
2019	3	33
2020	1	6
2021	0	0
2022	7	199
2023	4	74

Source: https://www.refugeecouncil.org.au/asylum-boats-statistics/.

Concern about the welfare and human rights of people held in immigration detention centres and scandals such as a wrongful detention, and a wrongful repatriation, of Australian citizens stoked community unease and protest against the hardline policies.^4^ Upon coming to power in November 2007, the Rudd Government ended offshore processing and Temporary Protection Visas.^3^ Boat arrivals subsequently increased each year (see Table 1). In 2010, the Gillard Government contemplated the unviable options of a regional processing centre in East Timor and an asylum seeker-refugee swap deal with Malaysia. Following recommendations by an expert panel, offshore processing was reinstituted in August 2012.^3^

On 19 July 2013, the Rudd Government announced that no person arriving by boat would be eligible for an Australian visa.^5^ Those previously sent offshore were returned to Australia, creating a peak of 10,201 people in onshore detention.^6^ There were 3129 adults and children sent to Manus Island and Nauru between July 2013 and mid-2014.^7^

What followed was a decade of increasing concern by Australians for the welfare of people in offshore detention. Mental and physical health became a point of tension as people deemed to require healthcare unavailable offshore were potentially eligible for transfer to Australia for treatment.^8^ Once physically present in Australia, legal measures may be instituted to prevent return offshore.

In early 2019, the Medevac Bill was passed to allow people on Nauru or Manus Island to transfer to Australia for medical treatment upon the recommendation of two independent Australian doctors.^8^ Under the legislation, 192 people were transferred to Australia before its repeal in late 2019.^9^ All children were removed from offshore by late February 2019.^10^ Following completion of a US resettlement program, all remaining willing transferees in Nauru were flown to Australia by June 2023.^11^ Subsequent boat arrivals have, however, led to a continuation of offshore processing on Nauru.^12^

Meanwhile, legislative amendments to the *Migration Act 1958 (Cth)* in late 2014 introduced mandatory visa cancellation for people without Australian citizenship who were considered not to pass ‘the character test’ outlined in Section 501 of *the Act*.^13^ This may occur, for example, following a conviction for a sexual offence against a child or if an individual receives a custodial sentence ≥12 months.^13^ From 2013 to 2016, the number of such cancellations increased sixteen-fold.^14^ The proportion of people held in onshore detention due to character-related visa cancellations increased from 33% in 2016 to 60% from 2021 onwards.^15^ Accordingly, most people now enter immigration detention directly from prison. Each individual must decide to either repatriate or undertake a lengthy administrative and legal process to regain their visa. Appeals can be made via the Administrative Appeals Tribunal, with judicial reviews by the Federal Court and the High Court, in a process that can last many years. The Administrative Review Tribunal replaces the AAT in October 2024.

In November 2023, the High Court of Australia (NZYQ v Minister for Immigration) determined that the constitution does not allow the government to continue to detain people in immigration detention where there is no real prospect of repatriation.^16^ This may occur, for example, when an individual deemed to not pass ‘the character test’ is stateless or came to Australia as a UNHCR refugee and is therefore not eligible to be repatriated due to Australia’s ‘non-refoulement’ obligations. A subsequent High Court decision allowed the ongoing detention of people not cooperating with repatriation.^17^ This may occur, for example, if a person from Iran is found to be not owed protection but is unwilling to return to Iran, as Iran is only willing to accept voluntary, not involuntary, repatriations.

## RANZCP advocacy

Over the last decade, the RANZCP has made public statements advocating for the following:

- The removal of all children and families from immigration detention^18^
- Detention not to be used for immigration processing^19^
- No mandatory detention for people who arrive by boat^20^
- Detention facilities only to be located onshore, and not in rural locations^20^
- No children, pregnant women, people with mental illness/disability or a history of torture or trauma, to be held in immigration detention^20^
- Psychiatrists treating people in detention to advocate for their release or to have their immigration determination expedited on mental health grounds^21^
- Increases to Australia’s Humanitarian Refugee Intake^22^

RANZCP’s position on these issues broadly aligns with the RACP’s 2015 Refugee and Asylum Seeker Health Position Statement which was endorsed by twelve other Australian health institutions, including the RACGP, various nursing bodies and a medical student association.^23^

In the March 2024 update of Position Statement 46, the RANZCP commended the decision of the High Court in NZYQ v Minister for Immigration.

## Potential issues with RANZCP’s position in this complex area

In RANZCP Position Statement 46, the college continues to advocate for changes to elements of Australia’s border protection system. This is recommended on the grounds of ‘a strong evidence basis showing that immigration detention can cause harm to physical and mental health’ and that ‘Self-harm and suicidal behaviour and ideation by immigration detainees is well-documented’. The references cited to support these assertions include six Australian Human Rights Commission sources (reports or transcripts of public hearings) published between 2013 and 2015, two United Nations reports published in 2014 and 2015, an Amnesty International report published in 2016, and a defunct online link to a 2015 systematic review noted to be last accessed by the Position Statement authors in 2016. When accessed through other means, the systematic review was found to conclude: ‘There is some evidence to suggest an independent adverse effect of detention on the mental health of asylum seekers. The conclusions should however be interpreted with caution as they are based on few studies. More research is needed in order to fully investigate the effect of detention on mental health’.^24^

In the Position Statement, the RANZCP does not address the complexity of obtaining reliable estimates of harm to mental health using measures with face validity in a setting where the endorsement of mental health symptoms may be perceived an advantage. Studies in this area do not address the overlap of genuine dissatisfaction/distress/despair regarding an immigration situation with high prevalence mental disorders including adjustment, mood and anxiety disorders. A reliance on statements of suicidality and self-harm as indices of mental illness or risk similarly needs to be acknowledged as occurring within a context, and possibly occurring for communicative purposes. An advocacy focus predicated on these metrics risks reinforcing suicidal statements and acts of self-harm as currency in the pursuit of immigration outcomes. It is important to note that statistics on completed suicide for people in detention are not available and mental health research in this area can only be conducted with government approval.

The RANZCP’s past advocacy for the removal of people with mental illness from detention did not include a discussion of individual variations in the expression of distress and mental health symptoms, as well as the influence of resilience, attachment style, culture and contextual factors in the communication of suffering. This is important to contemplate to avoid the injustice of giving priority to those most vocal and help-seeking but not necessarily most suffering. In addition, the RANZCP has not acknowledged the risks inherent in selecting groups, such as children, for improved treatment; for example, the unintended incentivising of the arrival of unaccompanied minors, or for adults to bring their own or other people’s children on the perilous journey.

Ultimately, Position Statement 46 sidesteps discussion of a central quandary: should the RANZCP, and RANZCP psychiatrists, advocate for fulfilment of the immigration goals of asylum seekers and people who have been deemed not to pass ‘the character test’ as a form of mental illness prevention or mental health treatment?

The answer to this question has real-world implications: Many RANZCP psychiatrists write psychiatric reports advocating for people to be transferred to Australia from offshore, or released from onshore immigration detention, on the grounds that it would improve the person’s mental health. Such reports are usually based on the subject’s self-report and written in the absence of independent collateral information. Position Statement 46 fails to provide guidance to psychiatrists writing such reports. Specifically, what is the minimum data set required, and what metrics or tools should psychiatrists use, to synthesise the complex cultural, forensic, traumatic and mental health factors, as well resilience factors, into a formulation that necessitates a particular person be prioritised above others for a more favourable immigration pathway?

## A broadening of the health advocate role

The concept of ‘health advocacy’ has broadened due to the growing awareness of the social determinants of health. According to the WHO, social determinants of health are ‘non-medical factors that influence health outcomes’.^25^ This includes, ‘The conditions in which people are born, grow, work, live, and age, and the wider set of forces and systems shaping the conditions of daily life’. Most broadly: ‘These forces and systems include economic policies and systems, development agendas, social norms, social policies and political systems’.^25^

The RANZCP Code of Ethics Statement 11.3 limits psychiatrist advocacy to ensuring that the best attainable mental health care is available. However, the CanMEDS criteria, which the RANZCP has endorsed as the curriculum framework for CPD requirements, expands the role of ‘health advocate’ to advocacy for patients ‘beyond the clinical environment’ and advocacy for communities and populations ‘for system-level change in a socially accountable manner’.^26^

Such broadening of understanding in the factors underpinning good mental health potentially pushes psychiatrists to take a political standpoint and political action, regarding the vexed question of what societal policies allow for the greatest mental health for the greatest number of people. In relation to Australia’s immigration policies, the RANZCP appears to have resolved this complex question in favour of seeing the greatest mental health likely to stem from reducing barriers to full community rights for asylum seekers as well as for people who have had their visa revoked on character grounds. While the College’s reasoning is unexplained, the perspective possibly reflects a view that compassion for excluded people is the highest ethical principle. This may reflect the RANZCP adhering to a singular narrative that all asylum seekers are fleeing their country in fear for their life. The grounds for the RANZCP adopting a singular narrative need to be explicated. To not do so risks the RANZCP appearing reluctant to acknowledge the complexity of rational human beings and the modern world, with a decision to transit to Australia potentially related to various economic, relationship, experiential, criminal and aspirational reasons, as well as other complex push and pull factors. Similarly, RANZCP’s expressed support for the High Court’s NZYQ v Minister for Immigration decision warrants greater discussion of the tension between: compassion, justice, individual rights, victim’s rights and community protection.

To maintain credibility with the Australian public on this issue, RANZCP’s Position Statement 46 ought to acknowledge the intense international debate regarding the community impact of absorbing a large number of asylum seekers and refugees. There are over 100 million displaced persons, asylum seekers and refugees worldwide.^27^ Such people settled in a peaceful and prosperous country may be highly motivated to succeed and may bring a diverse perspective and valuable skills, thereby enriching Australia. Community concerns, however, regarding the impact of a high proportion of asylum seekers and refugees in the community include: pressure on housing, jobs, infrastructure and health systems; crime risks, and barriers to integration and the formation of shared values.^28,29^ The RANZCP should address such concerns in a respectful and straightforward manner. Reliable statistics should be used to guide consideration.

## Conclusion

RANCZP Position Statement 46 reiterates the RANZCP’s longstanding position against immigration policies designed to deter boat arrivals and limit community access for people deemed to not pass ‘the character test’. This position appears to be aimed at preventing or treating mental illness in this group. However, the Position Statement lacks sufficient analysis of the complexities, and any unintended impacts on the community, of the RANZCP position. Reputational risks may accrue on the RANZCP from persistently advocating against current immigration policies without acknowledging the complexities of the issue and the concerns of the wider community.
